# Reduced Intensity transplantation vs chemotherapy in CR1. A prospective, pseudorandomized study in 50–70 year old AML patients

**DOI:** 10.1038/s41409-024-02408-x

**Published:** 2024-09-02

**Authors:** Mats Brune, Thomas Kiss, Harald Anderson, Malin Nicklasson, Robert Delage, Jürgen Finke, Tobias Gedde-Dahl, Josée Hébert, Martin Höglund, Ain Kaare, Vladimir Lazarevic, Lars Möllgård, Kari Remes, David Ritchie, Alexandros Spyridonidis, Mitchell Sabloff, Ruth Spearing, Elisabeth Wallhult, Per Ljungman

**Affiliations:** 1 Section of Hematology and Coagulation, Department of Specialist Medicine, Sahlgrenska University Hospital, Gothenburg, Sweden; 2 Hopital Maisonneuve-Rosemont, Division of Hematology, Oncology, Hematopoietic Cell Transplant and Cellular therapy, Université de Montréal, Montréal, QC Canada; 3 Dept. of Oncology Lund University, Lund, Sweden; 4 Hematology Department, Centre Hospitalier Universitaire, Laval University, Quebec, QC Canada; 5 Department of Internal Medicine I, Hematology, Oncology and Stem Cell Transplantation, Freiburg University Medical Center, Freiburg, Germany; 6 Department of hematology and Institute for Clinical Medicine, Oslo University Hospital, Oslo, Norway; 7 Dept. of Medical Sciences, Uppsala University Hospital, Uppsala, Sweden; 8 Department of Hematology and BMT, Tartu University Hospital, Tartu, Estonia; 9 Department of Hematology, Oncology and Radiation Physics, Skåne University Hospital, Lund, Sweden; 10 Dept. of Hematology, Turku University Hospital, Turku, Finland; 11 Bone Marrow Transplant Service, Clinical Haematology, Peter MacCallum Cancer Centre and Royal Melbourne Hospital, Melbourne, VIC Australia; 12 BMT Unit and Institute of Cell Therapy, University of Patras, Patras, Greece; 13 Division of Hematology, Department of Medicine, University of Ottawa and The Ottawa Hospital Research Institute, Ottawa, ON Canada; 14 Department of Haematology, Christchurch Hospital, Christchurch, New Zealand; 15 Dept. of Cellular Therapy and Allogeneic Stem Cell Transplantation, Karolinska Comprehensive Cancer Center, Karolinska University Hospital Huddinge, Div. of Hematology, Dept. of Medicine Huddinge, Karolinska Institutet, Stockholm, Sweden

**Keywords:** Stem-cell therapies, Acute myeloid leukaemia

## Abstract

The aim of this prospective, international multicenter, pseudorandomized study comparing RICT HCT to standard-of-care chemotherapy in intermediate- or high-risk AML patients 50–70 years using the *donor versus no-donor* concept. Part 1 included only patients with potential family donors (RD) at the date of HLA-typing of the first potential sibling or CR-date, if later. Part 2 allowed the inclusion of patients without a possible sibling donor using the start of an unrelated donor (URD) search as inclusion date. 360 patients were registered and 309 analyzed. The median follow-up was 47 months (1–168). There was no difference in overall survival (OS) between the RD (*n* = 124) and the Control (*n* = 77) groups (*p* = 0.50, 3-year OS RD: 0.41(95% CI; 0.32–0.50); Controls: 0.49 (95% CI; 0.37–0.59)). The main cause of death was relapse (67% RD; 88% Controls). In Part 2, the 3-year OS was 0.60 (95% CI 0.50–0.70) for URD-HCT (*n* = 86) and 0.37 (95% CI 0.13–0.62) for Controls (*n* = 20), respectively (*p* = 0.10). When analyzing transplanted patients (Part 2), the OS at 3-years was higher for URD-HCT than RD-HCT (0.67 (0.55–0.76) vs. 0.42 (0.26–0.57; *p* = 0.005). This study doesn’t support elderly HLA-identical siblings as donors for older AML patients undergoing a RICT allogeneic HCT in first CR.

## Introduction

The curative potential of allo-HCT is due to the preparative chemotherapy, and the immunological graft-versus-leukemia (GvL) effect exerted by donor T-cells. The introduction of reduced intensity and thereby lower toxicity conditioning before allo-HCT (RICT) has expanded the transplant option to elderly patients and patients with co-morbidities [1, 2], with the aim of retaining GvL whilst reducing non-relapse mortality (NRM).

Different approaches were used to compare allo-HCT with chemotherapy. These were mainly retrospective studies utilizing registry data [3–5]. A well-recognized problem is the difficulty to compare in a controlled setting allo-HCT vs. standard-of-care since stringent randomized studies are very difficult to perform. Imitating randomization, HCT vs chemotherapy studies have been performed using biological allocation (presence or absence of an HLA-identical sibling donor) in patients undergoing myeloablative HCT [6]. Zittoun et al found in a prospective study, where treatment allocation was done in first complete remission (CR1), an improvement after both auto- and allo-HCT as compared to chemotherapy [7]. In a similarly designed prospective study, Cassileth et al found a slight advantage of chemotherapy compared to auto- or allo-HCT [8]. In contrast, Cornelissen et al found an improvement in disease free survival by donor availability with transplants performed with myeloablative conditioning in patients <55 years having an intermediate or high-risk profile [9]. In this study, the aim was to use comparable starting points for the two treatments using the time for start of donor search for either a family or unrelated donor but also requiring that the patients had entered first complete remission.

## Methods

### Study design

The design of this study used the pseudorandomized donor versus no donor concept with the aim to compare RICT to standard-of-care chemotherapy in AML patients between 50 and 70 years. When the study was initiated (2003) only patients having a potential HLA-identical sibling (RD) were eligible for the study (Part 1). Due to an increased use of unrelated donors, the study design was changed to allow inclusion of patients without potential HLA-identical siblings (Part 2). Patients could be registered into the study at any time after diagnosis but were only eligible for study inclusion after having entered CR1. The date for study inclusion was the date the first sample was obtained for HLA-typing of a sibling (Parts 1 and 2) or start of unrelated donor (URD) search (Part 2). If either of these occurred before CR, the date of CR was set as the inclusion date. Patients never entering CR were excluded.

#### Study Part 1 (2003–2012)

The primary objective both for first part of the study and the whole study was to evaluate in an intention-to-treat setting the OS after RICT from HLA-identical sibling donors *compared to* standard-of-care chemotherapy. Additional objectives were to assess relapse-free survival (RFS), NRM, and relapse incidence (RI) between the two groups.

After entering CR1, patients with at least one sibling were informed about the study and after signed consent included into the study. If the sibling(s) were willing and had no contraindication for stem cell donation, HLA-typing was performed, and patients were assigned to one of two groups; RD (donor identified) or Controls (no donor).

#### Study Part 2 (2012–2016)

**Fig. 1:**
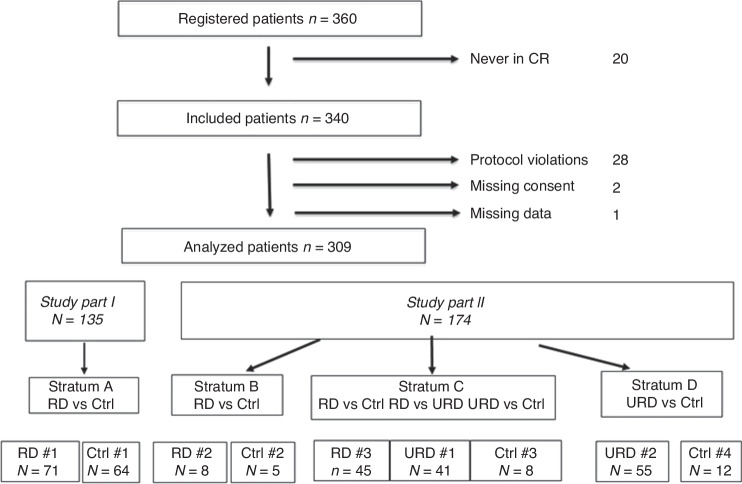
Study flow chart including reasons for exclusion from analysis. Flow chart describing patient inclusion and reasons for exclusions from analysis in the two different study periods.

The protocol was updated due to slow accrual and the increasing use of URD allowing search for a suitable URD *either* after failed RD search or upfront in patients without an available sibling. Depending on possible donor types, the patients were grouped into three strata (Fig. 1). HLA typing of potential related donors and unrelated donor search was permitted after registration but before confirmation of CR to decrease the time between diagnosis and transplant. Patients without a donor were allocated to one of the Control groups. The additional objectives of this part of the study, besides the original objective of comparing RD vs. Controls, were to assess if patients receiving RICT from URD had superior OS compared with Controls (no RD, no URD) or to RICT from RD. Other objectives were to analyze RFS, NRM, and RI after URD RICT compared to Controls.

### Study procedures

Patients ages 50–70 years with intermediate- or high-risk AML in CR1 and who were judged to be able to tolerate further therapy including RIC-HCT could be included. Important exclusion criteria were favorable risk AML as defined by cytogenetics and as of study part 2 by presence of mutation in NPM1 as sole molecular abnormality, abnormal renal (s-creatinine >2× ULN) or liver function (AST or ALT > 3× ULN), or severe concurrent illness preventing additional post-remission therapy.

### Treatments

Induction and consolidation therapy were given according to standard-of-care at participating centers, which varied between countries. The protocol recommended as conditioning regimens either the use of fludarabine 5–6 days (150–180 mg/sqm) combined with busulphan either given orally (total dose 8–11 mg/kg) or iv (Busulphex®; total dose 6.4 mg/kg) or a combination of fludarabine, carmustin, and melphalan. As graft-versus-host disease (GVHD) prophylaxis before URD HCT either anti-thymocyte globuline (ATG) or alemtuzumab was allowed. Recommended immunosuppression was ciclosporin (CyA) combined either with methotrexate (used in 63% of the patients) or mycophenolate mofetil (MMF).

### Cytogenetic and disease risk features

Two independent reviewers categorized the patients as high or intermediate risk according to the European Leukemia Net 2017 genetic risk classification [10, 11]. Interpretable reports from diagnostic bone marrow samples were available for 262 patients (85%). Patients with favorable risk cytogenetics were excluded including those with only NPM1 mutation.

Clinical high-risk features were defined as more than two inductions to reach CR1, secondary AML, and blasts ≥15% after first induction. Patients with any high-risk feature were assigned to the high-risk group. Risk factors in the different cohorts are presented in Supplementary table 1.

### Ethics approval

The study protocol and ensuing amendments were approved by the Ethical Committee at University of Gothenburg for Swedish patients (*S 240-04, S 288-03, S-266-03, S 272-03, S-231-12*), and by the corresponding authorities in participating centers and countries. All patients signed informed consent. The study was performed in accordance with all relevant regulations and guidelines in the participating countries.

### Administrative information

The study was registered at ClinicalTrial.gov (CTN #00342316).

### Statistics

Additional information about statistical analysis is given in Supplementary information.

The primary endpoint was OS in an intention to treat (ITT) setting. Baseline and treatment data were compared between ITT groups by Fisher´s exact tests and Wilcoxon´s rank-sum tests. Kaplan–Meier plots were used for illustration of time-to event endpoints. All endpoints (OS, RFS, NRM and RI) were determined from date of inclusion. The patients were grouped into strata A-D according to Fig. 1 based on the partition of the study in Part 1 and Part 2 and possible type of donor. In the ITT analyses, it was assumed that treatment allocation within strata was random. OS and RFS were compared by stratified log-rank tests and NRM and RI by stratified Gray´s tests considering competing risks. Three-year values with 95% confidence intervals are also presented. Hazard ratios (HR) were determined by means of stratified Cox regression. Proportions of patients with acute and chronic GVHD were analyzed with Fisher´s Exact test. Stata version 14 and R version 4.1.0 were used in the statistical analyses.

## Results

### Patient population and distribution

The study population is described in Fig. 1. 360 patients were registered into the study. Twenty patients never reached CR and were therefore not included. Thus, 340 patients were included in the study population. 31 were excluded due to donor search initiation before inclusion (*n* = 19), low-risk cytogenetics (*n* = 9), missing consent (*n* = 2), and missing data (*n* = 1). Moreover, three patients withdrew consent within the first three months after study inclusion. These are included in the analysis until date of consent withdrawal.

Thus, the analyzed study cohort included 309 patients of which 135 were included in Part 1 (2003–April 2012), and 174 in Part 2 (May 2012–2016) of the study. The age of patients (median 62 vs 64 years) was similar in the two parts. The median unrelated donor age was 25 (18–68) years (Part 2), whereas sibling donors were older; 60 (48–76) years; *p* < 0.001; Parts 1 and 2). The first patient was included December 18, 2003, and the last patient was included July 19, 2016. Data was analyzed as of August 1, 2018.

#### RD versus Controls; study parts 1 and 2 combined

The patient and donor distributions in the different study parts are shown in Fig. 1. 71 patients were assigned to the RD/donor and 64 to the Control/no donor group in Part 1 of the study (Fig. 1; Stratum A). Some study sites continued during the 2^nd^ part of the study including only patients fulfilling the criteria of study Part 1 (Stratum B: RD; *n* = 8, and Controls; *n* = 5).

In study Part 2, 45 patients had RD and patients without either RD or URD (*n* = 8) were considered as controls for both the RD and URD comparisons (Stratum C). Thus, the total number of patients with RD was 124 (71 + 8 + 45) and the number of Controls was 77 (64 + 5 + 8). In the Control group, 8/77 (8%) patients were treated off-protocol by allografting from alternative donors. These patients were included in the intention to treat (ITT) analysis as Controls.

#### URD versus Controls; study part 2

An URD was identified for 96 patients. Twenty patients without an identified donor were Controls to the URD group (Fig. 1; Strata C and D). Six patients underwent transplants from either cord blood or haploidentical donors. These patients were included as Controls in the ITT analysis. Two of these patients (from Stratum C) were controls also for the RD versus Controls comparison.

### RD versus Control (Part 1 and 2)

201 patients were included in the RD versus Control ITT comparison, 138 (69%) of whom died during follow-up. Median (min–max) follow-up time for the surviving patients was 76 (1–168) months. Patient characteristics were similar between RD and Control groups (Table 1). Ninety-seven patients underwent RICT at a median of 10 (3–33) weeks post inclusion while 27/124 (22%) patients did not reach transplant due to early relapse (*n* = 18), medical infirmity (*n* = 4), withdrawn consent (*n* = 2) or death (*n* = 3).

**Table 1:** Characteristics of patients in the RD vs Control primary endpoint analysis.

	Controls (*n* = 77)	RICT/RD (*n* = 124)	*p*-value
Gender F/M – *n* (%)	36 (47)/41 (53)	55 (44)/69 (56)	NS
Median age at Inclusion, years (min–max)	63 (51–70)	63 (51–71)	NS
Risk group IR/HR – *n* (%)	50 (65)/27 (35)	68 (55)/56 (45)	NS
Median time from diagnosis to inclusion, days (min–max)	64 (32–256)	64 (29–319)	NS
Given therapy			
Chemotherapy only	69	27	
Transplanted	8^a^	97^b^	
Median time from inclusion to transplant, days (min–max)		73 (23–236)	
Causes of death – *n* (%)			0.003
GvHD or related infection	–	9 (7)	
Infection	1 (1)	11 (9)	
Other	3 (4)	4 (3)	
Relapse	50 (65)	54 (44)	
Secondary malignancy	3 (4)	3 (2)	

^a^6 URD, 1 cord blood, 1 haploidentical HCT.

^b^All RICT/RD.

#### Overall survival

**Fig. 2:**
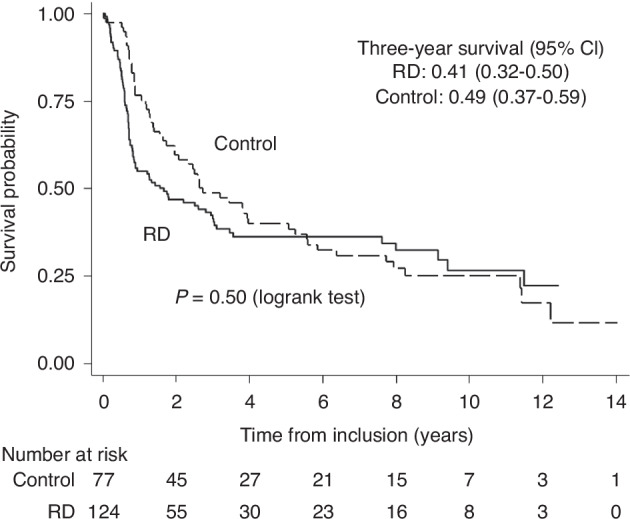
Overall survival from study inclusion in RD vs Controls. Kaplan-Meier estimates of 3-year survival in RD and control groups (study parts 1 and 2).

81/124 (65%) and 57/77 (74%) patients died in the RD and Control groups, respectively. Most deaths were due to relapse; 67% in the RD group and 88% in the Control group. Other causes are shown in Table 1. OS is shown in Fig. 2. At three years it was 0.41 (95% CI; 0.32–0.50) and 0.49 ((95% CI; 0.37–0.59); *p* = 0.50, logrank test) in the RD and control group, respectively. The data are not consistent with a constant hazard ratio over time: HR = 2.31 (95% CI: 1.34–4.01) before one year and HR = 0.57 (0.34–0.95) after one year (*p* = 0.0003 for difference) suggesting relatively higher mortality in the RD group the first year after inclusion and lower thereafter.

In an analysis performed with RD transplant as a time-dependent covariate, the analysis reflects the given treatment: A patient always starts as a Control patient and shifts to an RD patient at transplantation. The HR was 0.82 (0.56–1.18, *p* = 0.28) between the hazard for patients who received a transplant to the those who did not. Follow-up was censored at transplantation for the eight Controls transplanted off-protocol with alternative donors.

#### Other outcomes

*RFS:* 85/124 (69%) and 60/77 (78%) events occurred in the RD and Control groups, respectively (HR = 0.97 (0.69–1.37); *p* = 0.87). The RFS at 3 years was 0.38 (95% CI 0.29–0.46) in RD patients and 0.35 (95% CI 0.24–0.45) in Controls.

*NRM:* 27/124 (22%) and 7/77 (9%) non-relapse deaths occurred in the RD groups and Control groups, respectively. With relapse as a competing event, the cumulative NRM incidence at 3 years was 0.17 (0.11–0.25) in the RD group, and 0.039 (0.01–0.10) in Controls (*p* = 0.033, Gray’s test). *RI*: 58/124 (47%) and 53/77 (69%) patients relapsed in the RD and Control groups, respectively; With NRM as a competing risk, the cumulative relapse incidence at 3 years was 0.45 (95% CI 0.36–0.54) in the RD group, and 0.61 (95% CI 0.50–0.71) in Controls (*p* = 0.097, Gray’s test).

### Unrelated Donor (URD) *versus* No donor (Part 2)

*Results*. At study closure, 65/116 (56%) patients were alive after a follow-up of median 39 (3–70) months. 16/96 (17%) of the patients in the URD group did not undergo transplantation.

39/96 (41%) patients in the URD-group and 12/20 Controls (60%) died. Patient characteristics and causes of death are shown in Table 2.

**Table 2:** Characteristics of patients in the URD vs Control analysis.

	Controls (*n* = 20)	RICT/URD (*n* = 96)	*p*-value
Gender F/M – *n* (%)	10 (50)/10 (50)	36 (38)/60 (63)	NS
Median age at Inclusion, years (min–max)	63 (55–69)	64 (52–71)	NS
Risk group IR/HR – *n* (%)	10 (50)/10 (50)	46 (48)/50 (52)	NS
Median time from diagnosis to inclusion, days (min–max)	57 (32–160)	64 (28–244)	NS
Given therapy			
Chemotherapy only	14	16	
Transplanted	6^a^	80^b^	
Median time from inclusion to transplant, days (min–max)		93 (23–302)	
Cause of death – *n* (%)			NS
GvHD or rel inf	–	1 (1)	
Infection	1 (5)	5 (5)	
Other	0	3 (3)	
Relapse	10 (50)	30 (31)	
Secondary malignancy	1 (5)	0	

^a^1 cord blood, 5 haploidentical HCT.

^b^All RICT/URD.

**Fig. 3:**
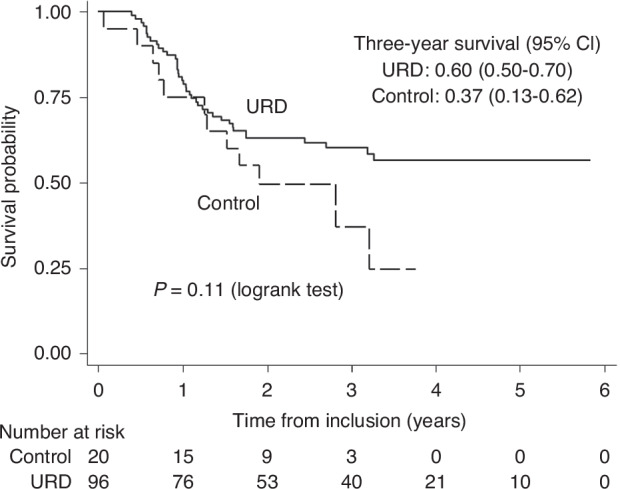
Overall survival from study inclusion in patients without potential sibling donor vs. controls. Kaplan-Meier estimated 3-year survival in patients without potential sibling donor (only study part 2).

The three-year OS was 0.60 (95% CI 0.50–0.70) in the URD-HCT group and 0.37 (95% CI 0.13–0.62) in the Control group (*p* = 0.10; Fig. 3). The HR comparing URD-HCT and Controls was 0.59 (95% CI 0.31–1.12; *p* = 0.11). There was neither any significant difference in RFS between URD-HCT and Controls (three-year values with 95% CI 0.52 (0.41,0.61) and 0.39 (0.19–0.60) respectively, *p* = 0.21), nor in NRM (0.10 (0.05–0.17) and 0.10 (0.02–0.27), *p* = 0.85), or RI (0.39 (0.29–0.48) and 0.51 (0.27–0.70), *p* = 0.32.

### RD versus URD versus Controls, according to given treatment (Part 2)

OS from study inclusion was analyzed with one time-varying covariates for each type of transplant. The HR´s were 0.70 (0.39–1.26) for RD-HCT versus Controls, and 0.27 (0.15–0.47) for URD-HCT versus Controls. A direct comparison yields that HR for URD-HCT was significantly lower than for RD-HCT (*p* = 0.0041) indicating that survival after URD was superior to RD.

### RD versus URD from transplantation (Part 2)

**Fig. 4:**
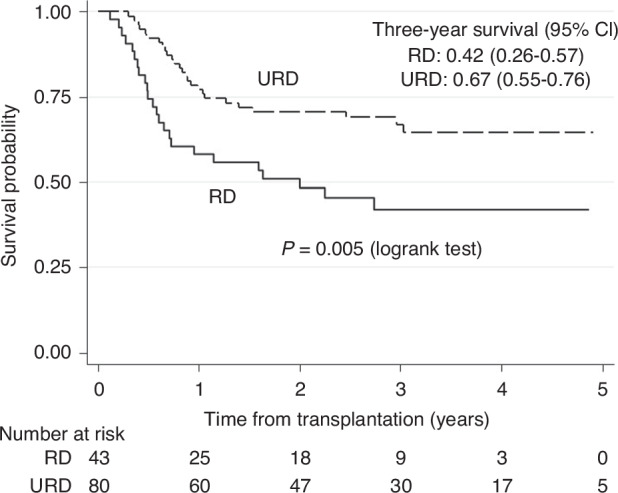
Overall survival in patients transplanted from unrelated or sibling donors. Kaplan-Meier estimates of 3-year survival in patients transplanted from URD or RD (only study period 2).

Patient characteristics are shown in Table 3. At study closure, 73 of 123 (59%) patients were alive after median 38 (1–66) months of follow-up from transplants. 24/43 (56%) patients died in the RD and 26/80 (33%) in the URD group. The OS was higher (*p* = 0.005) for patients undergoing URD (Fig. 4). Three year survival was 0.42 (0.26–0.57) in the RD-group and 0.67 (0.55–0.76) in the URD group. Furthermore, RFS was higher in the URD group (3-year RFS 0.61 [0.49,0.71] vs. 0.36 [0.21,0.51]; *p* = 0.01), NRM was lower (0.11 [0.05,0.19] vs. 0.30 [0.16,0.44]; *p* = 0.01), while there was no significant difference in RI (0.29 [0.19,0.39] vs 0.34 [0.20,0.49]; *p* = 0.46).

**Table 3:** Characteristics of RD vs URD patients in study period 2.

	RICT/RD (*n* = 43)	RICT/URD (*n* = 80)	*p*-value
Gender F/M – *n* (%)	18 (42)/25 (58)	30 (38)/50 (63)	NS
Median age at Inclusion, years (min–max)	63 (53–71)	65 (53–71)	NS
Time from CR to inclusion, days (min–max)	14 (0–173)	14 (0–138)	NS
Time from Inclusion to HCT days (min–max)	79 (23–236)	93 (23–302)	
Risk group IR/HR – *n* (%)	16 (37)/27 (63)	35 (44)/45 (56)	NS
Flu/Bu p.o./Not Flu/Bu p.o. – *n* (%)	16 (37)/27 (63)	50 (63)/30 (38)	0.008
No ATG/ATG – *n* (%)	33 (77)/10 (23)	5 (6)/75 (94)	<0.0001
Immunosuppression treatment – *n* (%)			0.008
CyA+MTX	22	61	
Other	21	19	
Donor age, median, yr (range)	61 (49–73)	25 (18–68)	<0.0001
Female donor to male recipient – *n* (%)			0.052
Yes	10 (23)	7 (9)	
No	33 (77)	73 (92)

### Acute and chronic GVHD in RD (Parts 1 + 2) and URD (Part 2)

There was a lower risk for acute GVHD grades III–IV in patients undergoing URD HCT (6% URD vs 19% RD; *p* = 0.01) and a lower risk for extensive chronic GVHD at 12 months (URD 12.7% vs. RD 40.3%; *p* < 0.001), 24 months (9.6% URD vs. 38.3% RD; *p* < 0.001), and 36 months (8.3% URD vs. 40% RD; *p* = 0.003) after transplantation compared to RD HCT patients (Supplementary Fig. 1).

## Discussion

Whether RICT allogeneic HCT in elderly patients with AML results in improved long-term survival has been discussed for many years. It has been exceedingly difficult to perform proper randomized prospective studies in allogeneic HCT in general due to the donor selection process including the willingness of patients and donors to be randomized to non-HCT therapy. Studies using other approaches have reported that RICT-HCT can result in long-term leukemia-free survival [1, 12–16]. However, a recent large prospective cohort study has challenged this concept in elderly patients and those with co-morbidities [17].

The aim of this study was to use pseudo-randomization based on the availability of at least one sibling willing to be typed and with the starting date either the day of a sample obtained for HLA-typing of a potential sibling donor or the day of CR if typing was performed before the patient entered CR. Our study, designed now several years ago, shows now with extended follow-up no advantage for a reduced intensity allogeneic-HCT with a sibling donor compared to Controls. As expected, the NRM was higher in the transplant group compared to Controls and although there was a slightly higher incidence of relapse in the Controls, this did not compensate for the higher risk for NRM. Similar results were seen when an URD was used, but the low number of Controls makes it difficult to draw any firm conclusion from this comparison. The main cause of death in all study groups was leukemia relapse and not NRM. Many relapses occurred in the “transplant groups” before the patient could get to transplant.

Several important advances in supportive care especially the introduction of new drugs against infections have occurred since the study was initiated improving the outcome of allogeneic HCT. Furthermore, the selected conditioning regimen has been shown to be inferior to more intensive conditioning regimens[10, 18], and it is possible that a more intensive but still reduced intensity regimen could have yielded better results. Interestingly, a large prospective study including patients during the latter part of our study period (2013–2017) also failed to show a survival benefit in elderly patients and in these who were medically infirm [17]. Moreover, new alternatives for non-transplant therapy of AML in the elderly have been introduced changing the therapeutic landscape [19]. The study included patients with a median age of 62–64 years in the different groups so not real elderly according to what is the clinical practice today. This, however, ought to have reduced rather than increased the risk for NRM if we translate the results to today’s situation.

The results of URD transplants were better than the results of HLA-identical sibling transplants with higher OS and RFS. Moreover, the NRM and the risks for especially severe acute and chronic GVHD were lower in the URD group suggesting that the best donor for an elderly patient might not be an HLA-identical sibling. This might be due to the positive impact of younger donor age on outcome of allo-HCT as shown in other studies [20–22]. Indeed, the median unrelated donor age was 25 years compared to 61 years in the RD group. It is important, however, that 94% of URD patients received ATG compared to only 11% of the patients receiving HLA-identical sibling donor transplants, which may explain the lower frequency of severe GVHD in the URD-group.

The strengths of this study are that it is large and multinational with analyses based on biological randomization enabling unbiased comparisons between HCT and Control. There are, however, also several weaknesses most importantly that many developments have occurred since the design of the study. Furthermore, the controls for the URD group were few making it difficult to draw conclusions from this comparison. Moreover, molecular risk stratification has not been evenly applied to study patients and Hematopoietic cell transplant comorbidity score data was not collected.

In summary, this study doesn’t support the use of elderly HLA-identical siblings as donors for older AML patients undergoing a RICT allogeneic HCT in first CR. It moreover indicates that a younger URD might be superior to an elderly RD.

## Supplementary information

Supplementary information

## Data Availability

The datasets generated during and/or analyzed during the current study are not publicly available due to patient and donor confidentiality but are available from the corresponding author on reasonable request.
