# Supplementary material for: Reduced Intensity transplantation vs chemotherapy in CR1. A prospective, pseudorandomized study in 50–70 year old AML patients
Source: Bone Marrow Transplant. 2024 Sep 2;59(12):1676–82. doi: 10.1038/s41409-024-02408-x (PMC11611731; doi:10.1038/s41409-024-02408-x)
Supplement: Supplementary file 1 — Supplementary information [file 41409_2024_2408_MOESM1_ESM.docx]

# Supplementary information

**Administrative information and data collection**

Patients were enrolled in nine countries: Sweden (n=161), Canada (n=94), Norway, (n=17), Australia (n=11), Finland (n=10), New Zealand (n=6), Greece (n=5), Estonia (n=3), Germany (n=2).

At inclusion, patient and disease characteristics were recorded in a Case Record Form. Relapse, GvHD, and adverse events were reported at 3, 8, and 12 months after inclusion, and then annually until death or end-of-study. On site data monitoring was performed at 7 centers (Montreal, Lund, Gothenburg, Umeå, Stockholm, Melbourne, Oslo).

**Statistics**

The sample size calculation was based on OS in the RD versus Control comparison. It was assumed that OS was 30% at three years in the Control group (Swedish AML-registry data) and that it would increase to 50% in the RD group, corresponding to a hazard ratio of 1.75. With equal number of patients in the two treatment arms, and using a log-rank test, the power was estimated at 90% to detect such a difference after 134 deaths have been reported.

The proportional hazards assumption was tested by means of Schoenfeld´s residuals. In the RD versus Control comparison of OS this assumption was strongly violated, and thus different HR´s were assumed before and after one year in an ad hoc Cox analysis. RD transplantation was also analyzed as a time-varying covariate in the RD vs. Control comparison of OS. In the 2nd part of the study, RD, URD, and a small number of haploidentical or cord blood transplantations were used in parallel, and the three different types of HCT were included as three different time-varying covariates in a Cox model with Control as reference. A direct comparison between RD and URD from date of transplantation was also performed.

**Risk factor reviewers:**

Josée Hébert (Québec, Canada), and Vladimir Lazarevic (Lund, Sweden).

**Supplementary table 1.** Detailed risk factor analysis for relapse in the different study cohorts:

|  | RICT/RD  n=124 | Control/RD  n=77 | RICT/MUD  n=96 | Control/MUD  n=20 |
| --- | --- | --- | --- | --- |
| Cytogenetic risk – n (%) | | | | |
| Low risk | 1 (1) | 0 | 0 | 0 |
| Intermediate risk | 79 (64) | 55 (71) | 66 (69) | 14 (70) |
| High risk | 23 (19) | 10 (13) | 18 (19) | 3 (15) |
| Adverse | 8 | 4 | 7 | 2 |
| Complex | 3 | 4 | 5 | 1 |
| Monosomy | 7 | 0 | 5 | 0 |
| Complex and monosomy | 5 | 2 | 1 | 0 |
| Other (F, U, UNK) | 9 (7) | 10 (13) | 7 (7) | 3 (15) |
| ND | 12 (10) | 2 (3) | 5 (5) | 0 |
| Risk group with FLT3 and NPM1* | | | | |
| Low risk | 1 (1) | 1 (1) | 0 | 0 |
| Intermediate risk | 29 (23) | 10 (13) | 62 (65) | 14 (70) |
| High risk | 5 (4) | 0 | 5 (5) | 2 (10) |
| ND | 89 (72) | 66 (86) | 29 (30) | 4 (20) |
| Clinical risk | | | | |
| Normal | 81 (56) | 57 (74) | 61 (64) | 14 (70) |
| High | 43 (35) | 20 (26) | 35 (36) | 6 (30) |
| s- or t-AML | 26 (21) | 12 (16) | 16 (17) | 4 (20) |
| Blast | 12 (10) | 8 (10) | 16 (17) | 2 (10) |
| s-or t-AML and Blast | 5 (4) | 0 | 3 (3) | 0 |
| Number of high risk factors | | | | |
| 1 | 39 (31) | 24 (31) | 41 (43) | 9 (45) |
| 2 | 14 (11) | 3 (4) | 8 (8) | 1 (5) |
| 3 | 3 (2) | 0 | 1 (1) | 0 |
| Total risk | | | | |
| Intermediate | 68 (55) | 50 (65) | 46 (48) | 10 (50) |
| High | 56 (45) | 27 (35) | 50 (52) | 10 (50) |

 * Only performed by some centers in part 2 of the study

**Supplementary figure 1.** Proportions av patients with related and unrelated donors and rates of chronic GVHD.

Patients at risk:
